# Dexmedetomidine Versus Dexamethasone in Pediatric Cardiac Surgery with Cardiopulmonary Bypass: An Exploratory Randomized Controlled Trial of Inflammatory Biomarkers and Opioid Consumption

**DOI:** 10.3390/healthcare14203468

**Published:** 2026-10-15

**Authors:** Mohamed A. Salama, Fatma K. Al Dammas, Abdullah AlDhuwaihy, Khalid Alanezi, Faisal Alshuwaier, Naif Almedlej, Hosam AbdelTwab, Noha ElTantawi, Rami A. Sabri, Ahmed Alaidy, Yahya F. Jamous

**Affiliations:** 1 Department of Anesthesia and Surgical Intensive Care, Faculty of Medicine, Mansoura University, Mansoura 35516, Egypt; 2 Department of Anesthesia, College of Medicine, King Saud University, Riyadh 11472, Saudi Arabia; 3 College of Medicine, King Saud University, Riyadh 11472, Saudi Arabia; 4 Department of Clinical Pathology, Faculty of Medicine, Mansoura University, Mansoura 35516, Egypt; 5 Department of Pediatrics, Faculty of Medicine, Mansoura University, Mansoura 35516, Egypt; 6 Department of Cardiothoracic Surgery, Faculty of Medicine, Mansoura University, Mansoura 35516, Egypt; 7 Wellness and Preventive Medicine Institute, Health Sector, King Abdulaziz City for Science and Technology (KACST), Riyadh 11442, Saudi Arabia

**Keywords:** dexmedetomidine, dexamethasone, pediatric cardiac surgery, cardiopulmonary bypass, interleukin-6, interleukin-8, inflammation, opioid sparing, fentanyl, children

## Abstract

**Background/Objectives:** Cardiopulmonary bypass (CPB) triggers a systemic inflammatory response in children undergoing congenital heart surgery. Although dexmedetomidine has been proposed as an alternative to corticosteroids, the two agents have not previously been directly compared in a pediatric randomized trial. This study aimed to compare the effects of dexmedetomidine and dexamethasone on inflammatory biomarkers and analgesic outcomes in children undergoing congenital heart surgery with CPB. **Methods:** In this prospective, randomized, controlled exploratory trial, 50 children aged 6 months to 18 years undergoing elective congenital heart surgery with CPB were randomized 1:1 to dexamethasone (*n* = 25) or dexmedetomidine (*n* = 25), using institutional regimens that were not equipotent anti-inflammatory doses. Serum interleukin-6 (IL-6) and interleukin-8 (IL-8) were measured at baseline, immediately after separation from CPB (post-CPB), and 12 h postoperatively. The primary endpoint was IL-6 at the two postoperative time points; IL-8 was a secondary biomarker outcome. Secondary outcomes also included Face, Legs, Activity, Cry, Consolability (FLACC) pain scores, fentanyl consumption, and time to first rescue analgesia. Effect estimates included Hodges–Lehmann (HL) median differences with 95% confidence intervals (CIs); a post hoc baseline-adjusted mixed-effects model of log-transformed concentrations served as a sensitivity analysis. Secondary and exploratory analyses were not adjusted for multiple comparisons. **Results:** All 50 randomized children were analyzed. Median IL-6 was lower in the dexmedetomidine group at 12 h (50 vs. 178 pg/mL; HL difference 86 pg/mL, 95% CI 59 to 144; *p* < 0.001); the post-CPB difference (46 vs. 77 pg/mL; HL difference 34 pg/mL, 95% CI 0 to 112; *p* = 0.045) was imprecise and not significant after post hoc Bonferroni correction for the two primary comparisons. In the post hoc baseline-adjusted sensitivity model, the ratio of geometric mean IL-6 concentrations (dexmedetomidine/dexamethasone) was 0.35 (95% CI 0.22 to 0.58; *p* < 0.001). Among secondary and exploratory outcomes, for which *p*-values are nominal, IL-8 was also lower post-CPB (159 vs. 381 pg/mL; *p* = 0.001) and at 12 h (120 vs. 311 pg/mL; *p* = 0.002). Dexmedetomidine was associated with lower intraoperative (83.0 ± 30.7 vs. 142.6 ± 51.7 µg; *p* < 0.001) and cumulative 24-h fentanyl consumption (53.8 ± 17.4 vs. 105.4 ± 32.8 µg; *p* < 0.001), analyzed in absolute units with incompletely documented intraoperative doses, and a longer time to first rescue analgesia (4.68 ± 0.73 vs. 1.84 ± 0.23 h; *p* < 0.001). Time to extubation did not differ significantly (median 2 vs. 1 h; *p* = 0.204). Intensive care unit length of stay, an exploratory outcome recorded in days, was distributed toward shorter stays with dexmedetomidine (median 2 [interquartile range 1–2] vs. 2 [2–3] days; *p* = 0.007). No pre-specified adverse events occurred in either group, although the trial was too small to exclude clinically important safety differences. **Conclusions:** In this exploratory trial of two non-equipotent institutional regimens, dexmedetomidine was associated with lower early postoperative IL-6 and IL-8 concentrations and lower opioid consumption than dexamethasone. These hypothesis-generating biomarker and analgesic signals do not demonstrate therapeutic superiority or patient-centered clinical benefit and require confirmation in larger, adequately powered multicenter trials.

## 1. Introduction

Congenital heart disease (CHD) is among the most common major congenital anomalies, with a pooled global birth prevalence of approximately 9 per 1000 live births [1]. A substantial proportion of affected children require surgical repair with cardiopulmonary bypass (CPB). Although operative mortality has declined substantially, postoperative morbidity, including neurodevelopmental impairment, remains an important and multifactorial concern [2]. The systemic inflammatory response to CPB is considered one contributing factor and is characterized by activation of nuclear factor-κB and release of cytokines such as interleukin-6 (IL-6) and interleukin-8 (IL-8) [3].

CPB induces a systemic inflammatory response when blood comes into contact with the synthetic surfaces of the extracorporeal circuit, leading to complement activation and a cascade of inflammatory mediators [4,5,6,7]. In children, the magnitude of this response has been associated with postoperative organ dysfunction and prolonged intensive care [8]. Contemporary pediatric data support these associations. In infants undergoing open-heart surgery, higher postoperative IL-6 and IL-8 concentrations were associated with acute kidney injury and prolonged ventilatory support [9], while an early postoperative increase in IL-8 was associated with low cardiac output syndrome in a prospective pediatric cohort [10]. Although these observational findings do not establish that lowering cytokine concentrations improves clinical outcomes, they support the continued use of IL-6 and IL-8 as pharmacodynamic markers of the inflammatory response to CPB. Consequently, several anti-inflammatory strategies have been investigated to attenuate this response.

Corticosteroids have been used for several decades to modulate inflammation associated with pediatric cardiac surgery, and their effects on inflammatory biomarkers are well documented. In a randomized, double-blind trial involving 29 children, dexamethasone administered before CPB produced an approximately eightfold reduction in IL-6 [11]. In a subsequent randomized controlled trial of children undergoing open-heart surgery, dexamethasone reduced early postoperative IL-6 and increased IL-10, whereas IL-8 did not differ significantly between groups [12]. Despite these biochemical effects, the clinical impact of corticosteroids on postoperative outcomes remains uncertain. A pediatric meta-analysis found that corticosteroids did not significantly reduce infection or mortality, although postoperative fluid balance was improved [13]. Similarly, a systematic review of 12 randomized trials involving 2209 children found only a non-significant reduction in all-cause mortality, although mechanical ventilation duration, low cardiac output syndrome, and reoperation were reduced at the cost of increased hyperglycemia and postoperative insulin use [14]. The large STRESS trial of methylprednisolone in 1263 infants likewise failed to demonstrate improved clinical outcomes and reported increased hyperglycemia [15]. In contrast, a 2024 meta-analysis of patients younger than 65 years reported a survival benefit [16], highlighting heterogeneity in the available evidence. Corticosteroid use is also limited by concerns regarding hyperglycemia and impaired wound healing.

Dexmedetomidine, a selective α2-adrenergic agonist [17], has emerged as a potential alternative because of its sedative, analgesic, and anti-inflammatory properties, as well as its reported neuroprotective effects in preclinical and clinical models [18,19]. In a pediatric randomized trial, intraoperative dexmedetomidine attenuated the CPB-associated increases in inflammatory mediators, including nuclear factor-κB, tumor necrosis factor-α, and IL-6, in children with CHD [20]. A systematic review and meta-analysis of seven randomized trials similarly found reductions in IL-6 and brain-injury biomarkers within 24 h of pediatric cardiac surgery [21]. Another systematic review of six pediatric trials reported reduced IL-6 in four of the five studies that measured it [22]. In adult cardiac surgery with CPB, a 2025 meta-analysis of nine randomized trials also reported significant reductions in IL-6 and tumor necrosis factor-α, together with improved oxygenation indices [23]. However, the evidence is not uniform. A 2025 randomized trial involving 100 adults undergoing coronary artery bypass grafting found that intraoperative dexmedetomidine at 0.5 µg/kg/h without a loading dose did not attenuate postoperative increases in IL-6 or other inflammatory markers [24]. Whether the anti-inflammatory effects reported in positive studies reflect direct immunomodulation or are secondary to sympatholysis and reduced surgical stress signaling therefore remains uncertain [25].

Corticosteroids and dexmedetomidine are both used in contemporary pediatric cardiac anesthesia, yet comparative evidence between them remains limited. Existing pediatric and adult studies have generally evaluated each agent against placebo or standard care rather than directly against one another, and, to our knowledge, no pediatric randomized trial has compared the two head-to-head. This evidence gap is clinically relevant because the two agents differ substantially in their pharmacological profiles and adverse effects. Corticosteroids are associated with hyperglycemia and impaired wound healing, whereas dexmedetomidine may cause bradycardia and hypotension. In addition, dexmedetomidine provides analgesic and sedative effects that corticosteroids do not, raising the possibility that it could simultaneously modulate inflammation and reduce perioperative opioid requirements. A direct comparison is therefore warranted to characterize their relative pharmacodynamic and analgesic effects.

This exploratory randomized controlled trial aimed to compare dexmedetomidine and dexamethasone with respect to CPB-induced inflammatory biomarkers and perioperative analgesic outcomes in children undergoing congenital heart surgery. We hypothesized that intraoperative dexmedetomidine would produce an early anti-inflammatory biomarker profile, assessed using IL-6 and IL-8, comparable to or better than dexamethasone while reducing perioperative opioid requirements.

## 2. Materials and Methods

### 2.1. Study Design and Participants

This prospective, randomized, controlled exploratory trial was conducted between July 2024 and March 2025. The study protocol was approved by the Institutional Review Board of Medical Research Ethics, Faculty of Medicine, Mansoura University, Mansoura, Egypt (approval code R.24.02.2513), and the trial was registered with the Pan African Clinical Trials Registry (PACTR202406581259613). Written informed consent was obtained from the parents or legal guardians of all participants before enrollment, and age-appropriate assent was obtained from older children and adolescents. The study was conducted in accordance with the Declaration of Helsinki and is reported according to the CONSORT 2025 statement [26]. Because no feasibility objectives (such as recruitment, protocol adherence, or acceptability) or progression criteria were pre-specified, the trial does not meet the definition of a pilot or feasibility trial in the CONSORT extension for randomized pilot and feasibility trials [27]; it is therefore described as an exploratory randomized controlled trial. Its sample size was calculated for the primary biomarker endpoint (Section 2.7), and its findings are intended to be hypothesis-generating. The completed CONSORT checklist is provided in the Supplementary Materials.

**Figure 1:**
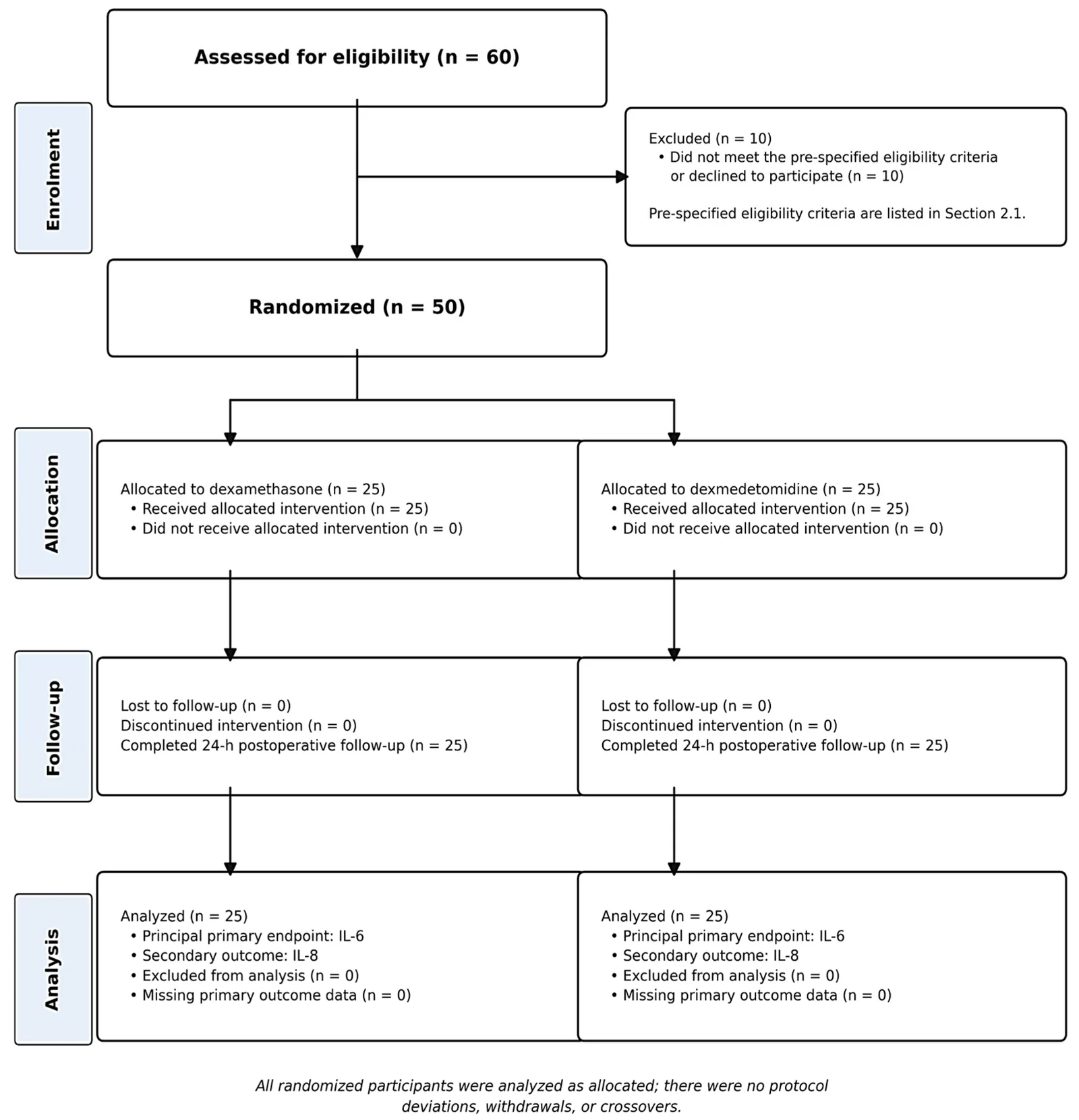
CONSORT 2025 flow diagram of participant enrollment, allocation, follow-up, and analysis. Of 60 children assessed for eligibility, 10 were excluded, and 50 were randomized 1:1 to dexamethasone or dexmedetomidine. All randomized participants received the allocated intervention and were included in the primary outcome analysis, with no loss to follow-up. IL-6 was the principal primary endpoint, and IL-8 was analyzed as a secondary biomarker outcome. Eligibility criteria are described in Section 2.1. IL, interleukin.

Eligible participants were children aged 6 months to 18 years who were scheduled for elective CHD repair requiring CPB and classified as American Society of Anesthesiologists (ASA) physical status II or III. Exclusion criteria included emergency surgery, redo sternotomy, pre-existing renal or hepatic dysfunction, irreversible or fixed pulmonary vascular disease, and known adrenal insufficiency. Flow-related reactive pulmonary hypertension associated with left-to-right shunts was not considered an exclusion criterion. Of the 60 children assessed for eligibility, 10 were excluded and 50 were enrolled and randomized. Participant flow is presented in the CONSORT diagram (Figure 1).

### 2.2. Randomization, Allocation Concealment, and Blinding

Participants were randomized in a 1:1 ratio to either the dexamethasone or dexmedetomidine group using a computer-generated random allocation sequence with fixed permuted blocks of four. The allocation sequence was generated and the allocation envelopes were prepared by an investigator who was not involved in patient care, outcome assessment, or data collection. Participants were screened and enrolled by the attending pediatric anesthesiologist, while treatment allocation and administration were performed by a member of the intraoperative anesthesia team. Allocation was concealed using sequentially numbered, opaque, sealed envelopes that were opened only after induction of anesthesia.

Because fixed block sizes may theoretically increase allocation predictability toward the end of each block, this represents a potential limitation of the randomization procedure. However, the use of sequentially numbered, opaque, sealed envelopes opened only after induction was intended to minimize the risk of selection bias.

Because the two interventions differed in their modes of administration, the intraoperative anesthesia team could not be fully blinded to treatment allocation. Personnel responsible for postoperative data collection, outcome assessment, and laboratory analyses were blinded to group assignment, as were the investigators performing the statistical analyses. To reduce the potential influence of unblinded intraoperative care, volume- and appearance-matched placebo preparations were administered at the time points corresponding to the comparator regimen.

### 2.3. Interventions

#### 2.3.1. Dexamethasone Group

Patients in the dexamethasone group (*n* = 25) received intravenous dexamethasone at 1 mg/kg per dose, with a maximum dose of 12 mg, at induction of anesthesia, at initiation of CPB, and 6 h after CPB, resulting in a cumulative dose of up to 3 mg/kg. This regimen reflected the institutional perioperative corticosteroid protocol and corresponded to the three-dose regimen (1 mg/kg, maximum 12 mg, at induction of anesthesia, at initiation of CPB, and 6 h later) evaluated in a previous pediatric randomized trial, in which it reduced postoperative IL-6 [12]; a single 1 mg/kg dose given after induction had also reduced IL-6 in an earlier randomized trial [11]. The dosing schedule was designed to cover the major inflammatory periods associated with induction, initiation of CPB, and early reperfusion. Blood glucose concentrations were monitored throughout the perioperative period, and insulin was administered according to the institutional protocol when glucose exceeded 180 mg/dL; these measurements were part of routine clinical care, and peak glucose concentrations, hyperglycemic episodes, and insulin use were not systematically recorded as study outcomes. Patients in this group also received a volume- and appearance-matched 0.9% saline placebo infusion from after induction until skin closure.

#### 2.3.2. Dexmedetomidine Group

Patients in the dexmedetomidine group (*n* = 25) received an intravenous loading dose of dexmedetomidine 0.5 µg/kg over 10 min after induction, followed by a continuous infusion of 0.1 µg/kg/h until skin closure. This regimen was based on a previously published randomized trial in children with CHD undergoing CPB, in which the same loading dose and infusion rate constituted the lower of two dexmedetomidine dose regimens evaluated; both regimens attenuated the increases in nuclear factor-κB, tumor necrosis factor-α, and IL-6 compared with saline, and the lowest marker levels were reported with the lower-dose regimen [20]. The infusion was discontinued at skin closure to minimize the potential for delayed extubation. At the time points corresponding to dexamethasone administration, patients received volume-matched 0.9% saline placebo injections.

The two regimens were selected to reflect the doses and schedules used clinically at the study institution for their respective indications, and each was consistent with dosing used in previous pediatric randomized trials of the corresponding agent [11,12,20]. The trial therefore compared the two agents as they are used in practice rather than at doses matched for anti-inflammatory activity. The regimens were not pharmacologically equivalent and could not be made so: dexamethasone is a long-acting glucocorticoid that suppresses inflammatory gene transcription through glucocorticoid receptors, whereas dexmedetomidine is a selective α2-adrenergic agonist with sympatholytic, sedative, and analgesic effects [17] whose anti-inflammatory action may be partly indirect [25], and no accepted dose equivalence exists between the two drug classes. The regimens also differed in the timing and duration of drug exposure: dexamethasone was administered at three time points, the last 6 h after CPB, whereas dexmedetomidine was administered only intraoperatively and was discontinued at skin closure. The study was therefore not designed to compare equipotent or maximally anti-inflammatory doses, and the findings should be interpreted as a comparison between two clinically used regimens rather than between the maximal pharmacological effects of the two agents.

### 2.4. Anesthetic and Surgical Management

A standardized anesthetic and surgical protocol was applied to all participants. Anesthesia was induced with sevoflurane in an oxygen–air mixture, fentanyl 5 µg/kg, and atracurium 0.4 mg/kg. Anesthesia was maintained with isoflurane at 0.8–1.2 minimum alveolar concentration in an air–oxygen mixture, a fentanyl infusion of 2–4 µg/kg/h, and supplemental atracurium as required.

Additional fentanyl boluses of 1–2 µg/kg were administered when clinical evidence of inadequate analgesia or a sympathetic response was observed. A sympathetic response was defined as an increase in heart rate or mean arterial pressure of >20% above baseline in the absence of hypovolemia, surgical artifact, or vasoactive-drug effects. Because dexmedetomidine can reduce heart rate, decisions regarding supplemental fentanyl administration in the dexmedetomidine group were guided primarily by mean arterial pressure and the surgical context rather than heart rate alone.

CPB was performed using a membrane oxygenator (Affinity Pixie, Medtronic, Minneapolis, MN, USA) and a blood-based prime consisting of packed red blood cells, fresh frozen plasma, and crystalloid as required, with a target hematocrit of at least 30%. Mild hypothermia at 30–32 °C was used. Antegrade blood cardioplegia at a 1:4 ratio was administered every 30 min, and pump flow was maintained at 2.4–3.2 L/min/m^2^. Mean arterial pressure during CPB was targeted at 40–60 mmHg according to age, with norepinephrine administered when required. Following separation from CPB, inotropic support consisted of milrinone at 0.8 µg/kg/min, supplemented with epinephrine at 0.1 µg/kg/min when necessary. Heparin was reversed with protamine at 4 mg/kg.

Postoperatively, all children were managed in the intensive care unit (ICU), where routine analgesia consisted of intravenous paracetamol 15 mg/kg every 6 h in both groups. Pain was assessed using the Face, Legs, Activity, Cry, Consolability (FLACC) scale [28] at 1, 2, 6, 12, 18, and 24 h postoperatively by personnel blinded to group allocation. Rescue analgesia with intravenous fentanyl boluses of 1–2 µg/kg was administered when the FLACC score was ≥4. Time to first rescue analgesia was defined as the interval from ICU admission to the first rescue dose, and cumulative fentanyl consumption was recorded over the first 24 postoperative hours.

### 2.5. Laboratory Measurement of IL-6, IL-8, and Cortisol

Venous blood samples for IL-6 and IL-8 measurement were obtained at three predefined time points: after induction of anesthesia and before skin incision (baseline), immediately after separation from CPB (post-CPB), and 12 h postoperatively. Serum cortisol was measured at baseline and 1 h postoperatively.

Blood samples were allowed to clot and were subsequently centrifuged. The separated serum was aliquoted and stored at −80 °C until batch analysis at the end of the study. Samples from both treatment groups were analyzed together in the same batch to minimize potential inter-assay variation. Laboratory personnel performing and interpreting the assays were blinded to treatment allocation.

Serum IL-6 and IL-8 concentrations were determined using quantitative sandwich enzyme-linked immunosorbent assays (ELISAs). IL-6 was measured using the Human IL-6 ELISA Kit (Catalogue No. EH0201; Wuhan Fine Biotech Co., Ltd., Wuhan, China), with a stated detection range of 4.688–300 pg/mL, a lower limit of detection of 2.813 pg/mL, an intra-assay coefficient of variation (CV) of <8%, and an inter-assay CV of <10%. IL-8 was measured using the Human IL-8 ELISA Kit (Catalogue No. EH0205; Wuhan Fine Biotech Co., Ltd., Wuhan, China), with a detection range of 7.813–500 pg/mL, a lower limit of detection of 4.688 pg/mL, an intra-assay CV of <8%, and an inter-assay CV of <10%.

ELISA kits and reagents were stored at 2–8 °C according to the manufacturer’s instructions. Samples were initially assayed without dilution. Samples with concentrations exceeding the upper limit of the calibration range were reassayed after dilution using the supplied sample diluent (1:2 for IL-6 and 1:4 for IL-8), and the measured concentrations were multiplied by the corresponding dilution factor. All samples were analyzed in duplicate, and the mean of the two measurements was used for statistical analysis. IL-6 concentrations below the lower limit of detection were not replaced by the lower limit of detection; they were retained in the analysis dataset as recorded (minimum, 2 pg/mL). Because these were the lowest values in the dataset, assigning them the lower limit of detection would not have changed their ranks relative to quantifiable concentrations; nevertheless, such values, and values below the lowest calibration standard (4.688 pg/mL for IL-6), should be regarded as imprecise. Serum cortisol was measured using an automated chemiluminescent immunoassay on the Cobas e411 analyzer (Roche Diagnostics, Mannheim, Germany) according to the manufacturer’s standard operating procedures.

### 2.6. Outcome Measures

The primary outcome was serum IL-6 concentration measured post-CPB and 12 h postoperatively. Because the sample size calculation was based on the anticipated between-group difference in IL-6, IL-6 was the principal primary endpoint. A single primary time point was not pre-specified; the two postoperative IL-6 comparisons therefore constitute the primary comparisons, and multiplicity across them is addressed in Section 2.7. Serum IL-8 at the same time points was pre-specified as a co-primary biomarker outcome; however, because the study was not powered for IL-8, it is reported and interpreted as a secondary biomarker outcome. Baseline concentrations were obtained before surgery and served as baseline measurements for comparison and adjustment.

Pre-specified secondary outcomes included FLACC pain scores [28], intraoperative fentanyl consumption, cumulative fentanyl consumption during the first 24 postoperative hours, time to first administration of rescue analgesia, heart rate and mean arterial pressure at predefined intraoperative time points, and serum cortisol as a marker of the surgical stress response. Time to extubation and ICU length of stay were assessed as exploratory outcomes, for which the study was not specifically powered. ICU length of stay was recorded in days.

Safety outcomes included intraoperative bradycardia, defined as a heart rate below the 5th percentile for age requiring intervention, and hypotension, defined as a mean arterial pressure below the 5th percentile for age requiring fluid administration or escalation of vasoactive support [29]. Postoperative complications recorded in all participants included reintubation, postoperative nausea and vomiting, respiratory depression, pruritus, and neurological deficit.

### 2.7. Statistical Analysis

The sample size calculation was based on the between-group difference in IL-6 reported by Amanullah et al. [12]. Assuming a two-sided α of 0.05, 80% statistical power, and an effect size of 0.88, 22 participants were required per group. This number was increased to 25 participants per group to account for an anticipated attrition rate of approximately 10%. Statistical analyses were performed using SPSS version 25 (IBM Corp., Armonk, NY, USA); the Hodges–Lehmann estimates and post hoc sensitivity analyses were performed using SPSS version 27.

Normality of continuous variables was assessed using the Shapiro–Wilk test. Approximately normally distributed continuous variables were compared using independent-samples *t*-tests, whereas non-normally distributed variables were analyzed using the Mann–Whitney *U* test. Categorical variables were compared using the χ^2^ test or Fisher’s exact test, as appropriate. In accordance with CONSORT recommendations, baseline characteristics were summarized descriptively and were not subjected to significance testing; between-group differences are presented with 95% confidence intervals (CIs).

Treatment effects were reported as effect estimates with 95% CIs in addition to *p*-values. Unless otherwise stated, between-group differences are expressed as dexamethasone minus dexmedetomidine. For approximately normally distributed continuous variables, mean differences with 95% CIs were calculated using the pooled-variance *t*-distribution, and Hedges’ *g* was reported as the standardized effect size. For variables analyzed using the Mann–Whitney *U* test (IL-6, IL-8, FLACC scores, time to extubation, and ICU length of stay), the Hodges–Lehmann estimate of the median difference (location shift) with its distribution-free 95% CI was reported as the effect estimate; for IL-6 and IL-8, effect size *r* was also calculated as *z*/√*N*. Because FLACC is an ordinal scale, FLACC scores were summarized as medians with interquartile ranges (IQRs) for inferential comparison; means with standard deviations are shown in Figure in Section 3.3.1 for descriptive purposes only. Exact *p*-values are reported, except that values below 0.001 are reported as *p* < 0.001.

Variables assessed repeatedly over time, including IL-6, IL-8, FLACC pain scores, heart rate, and mean arterial pressure, were compared between groups separately at each predefined time point, as pre-specified. Because this approach does not account for within-subject correlation, post hoc longitudinal sensitivity analyses were performed. IL-6 and IL-8 were analyzed using linear mixed-effects models of log_10_-transformed concentrations at the two postoperative time points, with treatment group, time, the group-by-time interaction, and the log_10_-transformed baseline concentration as fixed effects and a random intercept for each participant; between-group differences were back-transformed and expressed as ratios of adjusted geometric means (dexmedetomidine/dexamethasone) with 95% CIs. Heart rate and mean arterial pressure were analyzed using linear mixed-effects models with treatment group, time, and the group-by-time interaction as fixed effects and a random intercept for each participant. A cumulative-logit ordinal regression of FLACC scores including group, time, and their interaction was also attempted but could not be validly estimated because of quasi-complete separation of scores between groups (at 2 h, all scores were 4–5 in the dexamethasone group and 1–2 in the dexmedetomidine group); its results are therefore not reported. These longitudinal analyses were not pre-specified and are interpreted as sensitivity analyses.

Because baseline IL-6 concentrations were imbalanced between groups and markedly right-skewed, baseline adjustment was performed on the log_10_-transformed scale within the mixed-effects model described above rather than by analysis of covariance on the untransformed scale. The primary analysis remained the unadjusted rank-based comparison, and the adjusted findings were interpreted as supportive rather than confirmatory.

Fentanyl consumption was analyzed in absolute units (µg), consistent with the available trial dataset. Weight-normalized fentanyl consumption (µg/kg) was not available for analysis, and this limitation is addressed in the Discussion. Because supplemental intraoperative fentanyl was administered by the unblinded anesthesia team partly in response to hemodynamic changes, a post hoc sensitivity analysis using multiple linear regression examined the between-group difference in intraoperative fentanyl consumption after adjustment for heart rate and mean arterial pressure measured after induction of anesthesia.

The primary comparisons were the two postoperative IL-6 comparisons (post-CPB and 12 h). A two-sided *p* < 0.05 was pre-specified as the threshold for statistical significance; in addition, to account for multiplicity across the two primary comparisons, a Bonferroni-adjusted threshold of 0.025 was applied as a post hoc sensitivity criterion. IL-8 and all other secondary and exploratory analyses were considered hypothesis-generating, no adjustment for multiple comparisons was performed for these outcomes, and their *p*-values, which are nominal, were interpreted descriptively.

## 3. Results

For each outcome, the pre-specified analysis is reported first; for repeated measurements, this consisted of separate between-group comparisons at each time point (Section 2.7). The linear mixed-effects models, the regression analysis of intraoperative fentanyl consumption, the attempted ordinal model for FLACC scores, and the Bonferroni-adjusted threshold for the two primary IL-6 comparisons were not pre-specified; these post hoc sensitivity analyses are labeled as such throughout and are presented after the corresponding pre-specified results.

### 3.1. Participant Flow and Baseline Characteristics

Sixty children were assessed for eligibility between July 2024 and March 2025. Ten children were excluded, and 50 were randomized in a 1:1 ratio to the dexamethasone group (*n* = 25) or the dexmedetomidine group (*n* = 25). All randomized participants received the allocated intervention, none discontinued treatment or were lost to follow-up, and all 50 participants were included in the analysis of the primary outcomes. Participant flow is shown in Figure 1.

Baseline demographic and perioperative characteristics are summarized in Table 1. The groups were similar with respect to age, weight, aortic cross-clamp time, and CPB duration, with the 95% confidence intervals for the corresponding between-group differences including zero. In accordance with CONSORT recommendations, no significance testing was performed for baseline characteristics. The overall distribution of surgical procedures was broadly similar between groups, although numerical imbalances were observed for some individual diagnoses, including a greater number of ventricular septal defect closures in the dexamethasone group (6 vs. 1) and a greater number of single-ventricle palliation procedures in the dexmedetomidine group (7 vs. 4).

### 3.2. Inflammatory Biomarkers: Primary (IL-6) and Secondary (IL-8) Outcomes

Baseline IL-6 concentrations were numerically higher in the dexamethasone group, although the observed ranges overlapped between groups (Table 2). In the pre-specified primary analysis (Mann–Whitney *U* test), median IL-6 was lower in the dexmedetomidine group than in the dexamethasone group at 12 h postoperatively (50 vs. 178 pg/mL; Hodges–Lehmann difference 86 pg/mL, 95% CI 59 to 144; effect size *r* = 0.68; *p* < 0.001). Post-CPB, median IL-6 was also lower in the dexmedetomidine group (46 vs. 77 pg/mL; Hodges–Lehmann difference 34 pg/mL, 95% CI 0 to 112; *r* = 0.28; *p* = 0.045); however, the lower confidence limit of 0 pg/mL indicates an imprecise estimate, and this comparison would not remain statistically significant at the post hoc Bonferroni-adjusted threshold of 0.025, whereas the 12-h comparison would.

Baseline IL-8 concentrations were comparable between groups. In the pre-specified analysis of the secondary IL-8 outcome, median IL-8 was lower in the dexmedetomidine group than in the dexamethasone group post-CPB (159 vs. 381 pg/mL; Hodges–Lehmann difference 160 pg/mL, 95% CI 76 to 267; *r* = 0.45; nominal *p* = 0.001) and at 12 h postoperatively (120 vs. 311 pg/mL; Hodges–Lehmann difference 159 pg/mL, 95% CI 37 to 244; *r* = 0.44; nominal *p* = 0.002). Because IL-8 was a secondary outcome and these *p*-values were not adjusted for multiple comparisons, the IL-8 findings are considered supportive and hypothesis-generating.

In post hoc sensitivity analyses that were not pre-specified (Section 2.7), baseline-adjusted linear mixed-effects models of log_10_-transformed concentrations gave results consistent with the pre-specified rank-based analyses. After adjustment for log_10_-transformed baseline IL-6, IL-6 concentrations across the two postoperative time points were lower in the dexmedetomidine group (ratio of adjusted geometric means 0.35, 95% CI 0.22 to 0.58; *F*(1, 47) = 17.58, *p* < 0.001), with no evidence that the between-group difference varied between the post-CPB and 12-h time points (group-by-time interaction *p* = 0.414). In the corresponding model for IL-8, the ratio of adjusted geometric means was 0.50 (95% CI 0.36 to 0.71; *F*(1, 47) = 15.85, *p* < 0.001; group-by-time interaction *p* = 0.986). These post hoc findings are interpreted as supportive rather than confirmatory.

**Figure 2:**
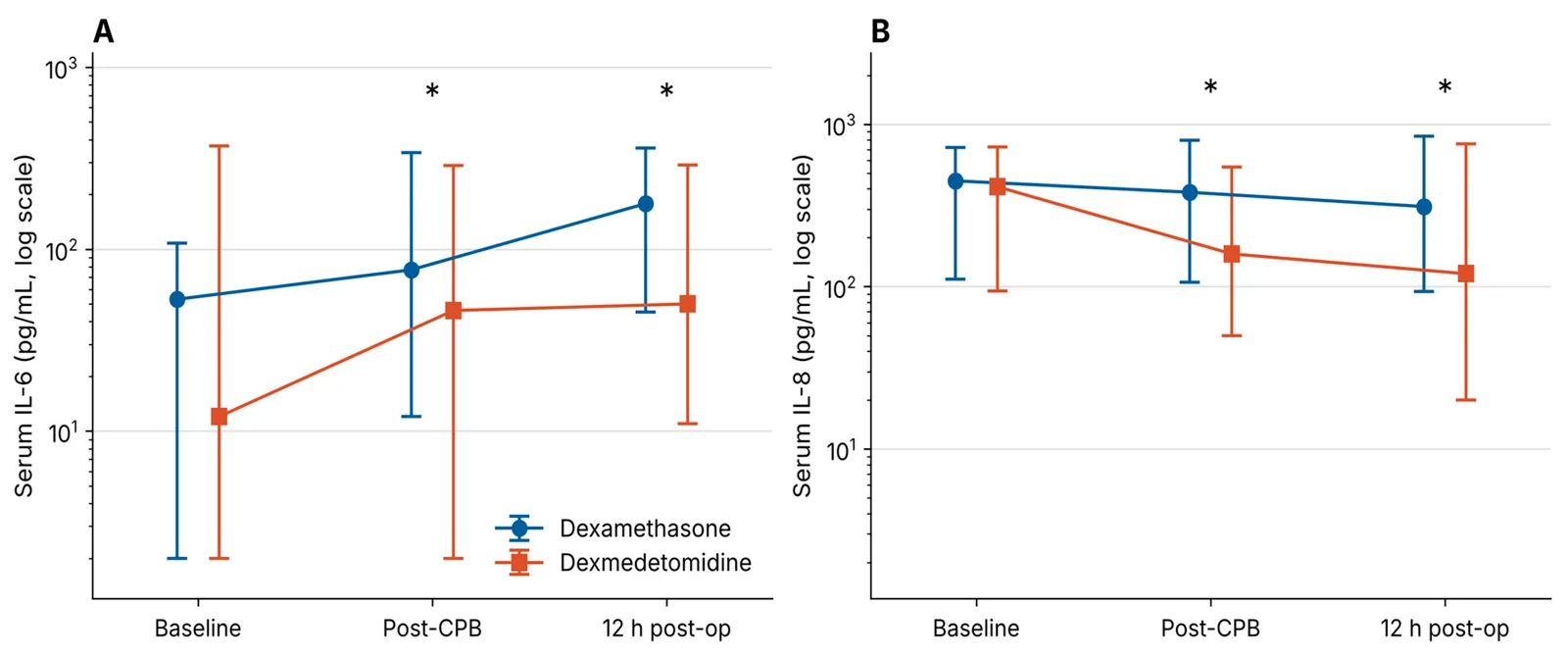
Serum interleukin concentrations by group and time point. (**A**) IL-6 and (**B**) IL-8 concentrations in the dexamethasone and dexmedetomidine groups at baseline, immediately after separation from cardiopulmonary bypass (post-CPB), and 12 h postoperatively. Symbols represent group medians, and vertical bars indicate the observed minimum-to-maximum range. The *y*-axis is presented on a logarithmic scale because both biomarkers were markedly right-skewed. Points were horizontally offset to improve visualization of the range bars. Asterisks indicate between-group comparisons with *p* < 0.05 using the Mann–Whitney *U* test (IL-6, primary outcome: *p* = 0.045 post-CPB and *p* < 0.001 at 12 h, of which only the 12-h comparison remains significant at the post hoc Bonferroni-adjusted threshold of 0.025; IL-8, secondary outcome not adjusted for multiplicity: nominal *p* = 0.001 post-CPB and *p* = 0.002 at 12 h). Baseline concentrations are presented descriptively and were not subjected to significance testing. All values are reported in Table 2. CPB, cardiopulmonary bypass; IL, interleukin.

The magnitude of the rank-based effect was largest for IL-6 at 12 h, whereas the post-CPB IL-6 effect was small to moderate and the IL-8 effects were moderate. Although the rank-based between-group separation in IL-6 appeared greater at 12 h than post-CPB, the post hoc longitudinal model provided no evidence that the IL-6 difference varied between the two time points; the magnitude of the IL-8 difference was similar at the two postoperative time points. Biomarker concentrations by group and time point are illustrated in Figure 2.

### 3.3. Secondary and Exploratory Outcomes

#### 3.3.1. Opioid Consumption and Analgesia

In the pre-specified comparisons, recorded intraoperative fentanyl consumption was lower in the dexmedetomidine group than in the dexamethasone group (83.0 ± 30.7 vs. 142.6 ± 51.7 µg), corresponding to a mean difference of 59.6 µg (95% CI 35.4 to 83.8), Hedges’ *g* = 1.38, and 42% lower mean recorded consumption (*p* < 0.001). Similarly, cumulative fentanyl consumption during the first 24 postoperative hours was lower with dexmedetomidine (53.8 ± 17.4 vs. 105.4 ± 32.8 µg), with a mean difference of 51.6 µg (95% CI 36.7 to 66.5), Hedges’ *g* = 1.93, and 49% lower mean consumption (*p* < 0.001) (Table 3). These comparisons are based on absolute recorded doses. Because documentation of intraoperative fentanyl was incomplete, the recorded intraoperative values underestimate total intraoperative exposure; the intraoperative mean difference should therefore be regarded as approximate, and the relative reduction is probably overstated (Section 4.5). Mean body weight was approximately 10% higher in the dexmedetomidine group (20.06 vs. 18.28 kg; Table 1), which, under weight-based dosing, would be expected to increase rather than decrease absolute consumption in that group; however, because weight-normalized consumption could not be analyzed, the observed differences may partly reflect differences in body size or dosing requirements. In a post hoc sensitivity analysis adjusting for heart rate and mean arterial pressure after induction of anesthesia, recorded intraoperative fentanyl consumption remained lower in the dexmedetomidine group (adjusted mean difference 63.8 µg, 95% CI 38.8 to 88.8; *p* < 0.001).

Time to first administration of rescue analgesia was longer in the dexmedetomidine group (4.68 ± 0.73 vs. 1.84 ± 0.23 h), corresponding to a mean difference (dexamethasone minus dexmedetomidine) of −2.84 h (95% CI −3.15 to −2.53; *p* < 0.001).

**Figure 3:**
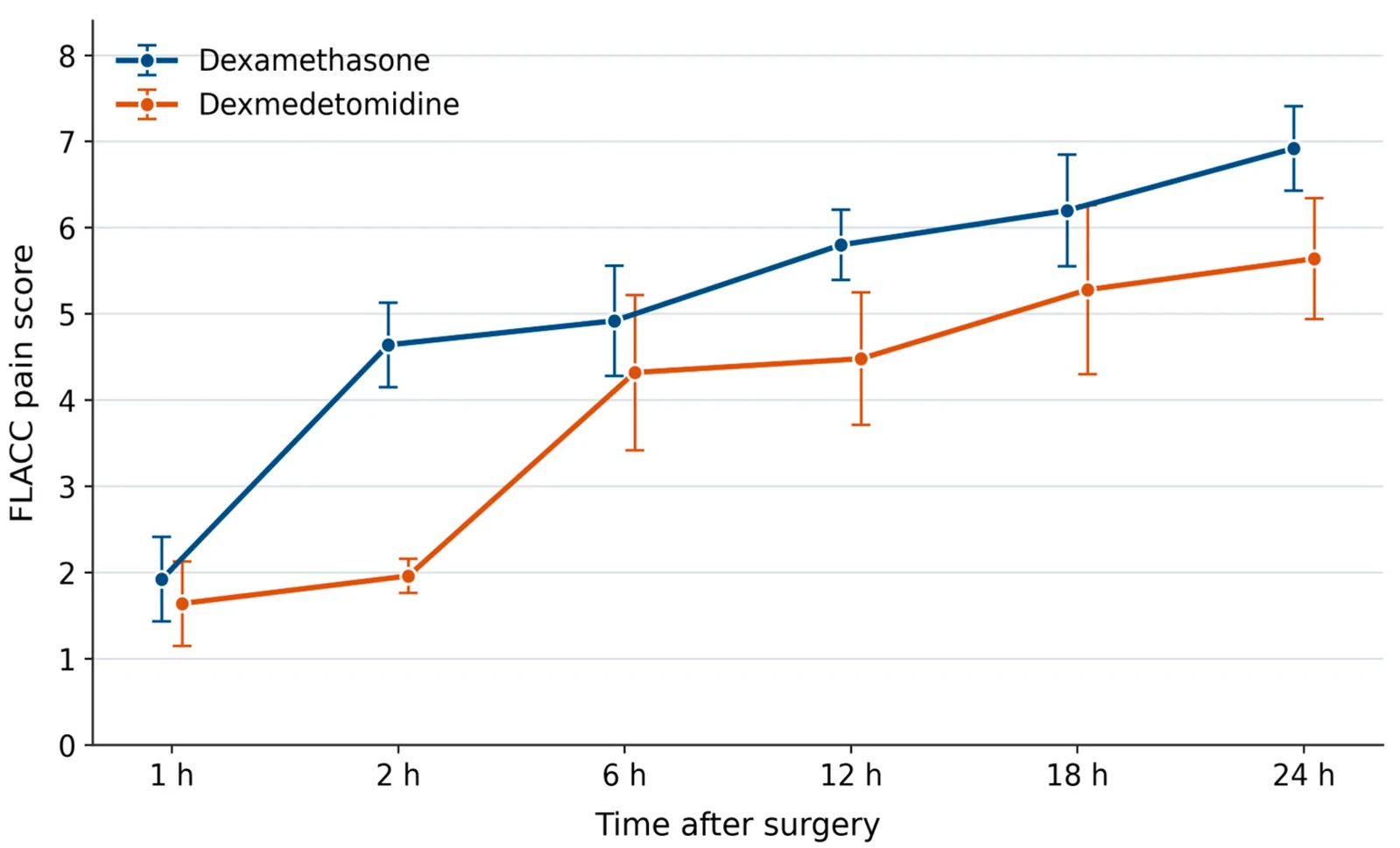
FLACC pain scores during the first 24 postoperative hours in the dexamethasone and dexmedetomidine groups. Data are presented as means with standard deviation error bars. Because FLACC is an ordinal scale, these values are presented as descriptive summaries; medians with IQRs, Hodges–Lehmann estimates, and Mann–Whitney *U* test results are reported in Table 3. Mean scores were numerically lower in the dexmedetomidine group at all measured time points and increased progressively in both groups over the first postoperative day. FLACC was a secondary outcome; the individual time-point comparisons were not adjusted for multiple comparisons, and the corresponding *p*-values are nominal and hypothesis-generating. Points were horizontally offset to improve visualization of the error bars. Full numerical data are provided in Supplementary Table S2. FLACC, Face, Legs, Activity, Cry, Consolability.

Mean FLACC pain scores were numerically lower in the dexmedetomidine group at all measured postoperative time points (Figure 3), and median scores were lower at all time points except 1 h (Table 3). In the pre-specified time-point comparisons, median scores did not differ significantly at 1 h (2 [IQR 1–2] vs. 2 [IQR 2–2]; Hodges–Lehmann difference 0, 95% CI 0 to 0; *p* = 0.057). The largest difference was observed at 2 h (2 [IQR 2–2] vs. 5 [IQR 4–5]; Hodges–Lehmann difference 3, 95% CI 3 to 3; *p* < 0.001), and lower scores persisted at 24 h (6 [IQR 5–6] vs. 7 [IQR 7–7]; Hodges–Lehmann difference 1, 95% CI 1 to 2; *p* < 0.001). Because FLACC was a secondary outcome compared at six time points without adjustment for multiple comparisons, these *p*-values are nominal and the FLACC findings are hypothesis-generating; a post hoc longitudinal ordinal model could not be validly estimated (Section 2.7). FLACC scores increased progressively in both groups over the first 24 postoperative hours despite rescue analgesia.

#### 3.3.2. Cortisol and Hemodynamic Outcomes

Serum cortisol concentrations were similar between groups at baseline (12.76 ± 2.05 vs. 13.56 ± 2.25 µg/dL) and did not differ significantly at 1 h postoperatively (31.72 ± 1.59 vs. 32.24 ± 2.70 µg/dL; mean difference 0.52 µg/dL, 95% CI −0.74 to 1.78; *p* = 0.411).

**Figure 4:**
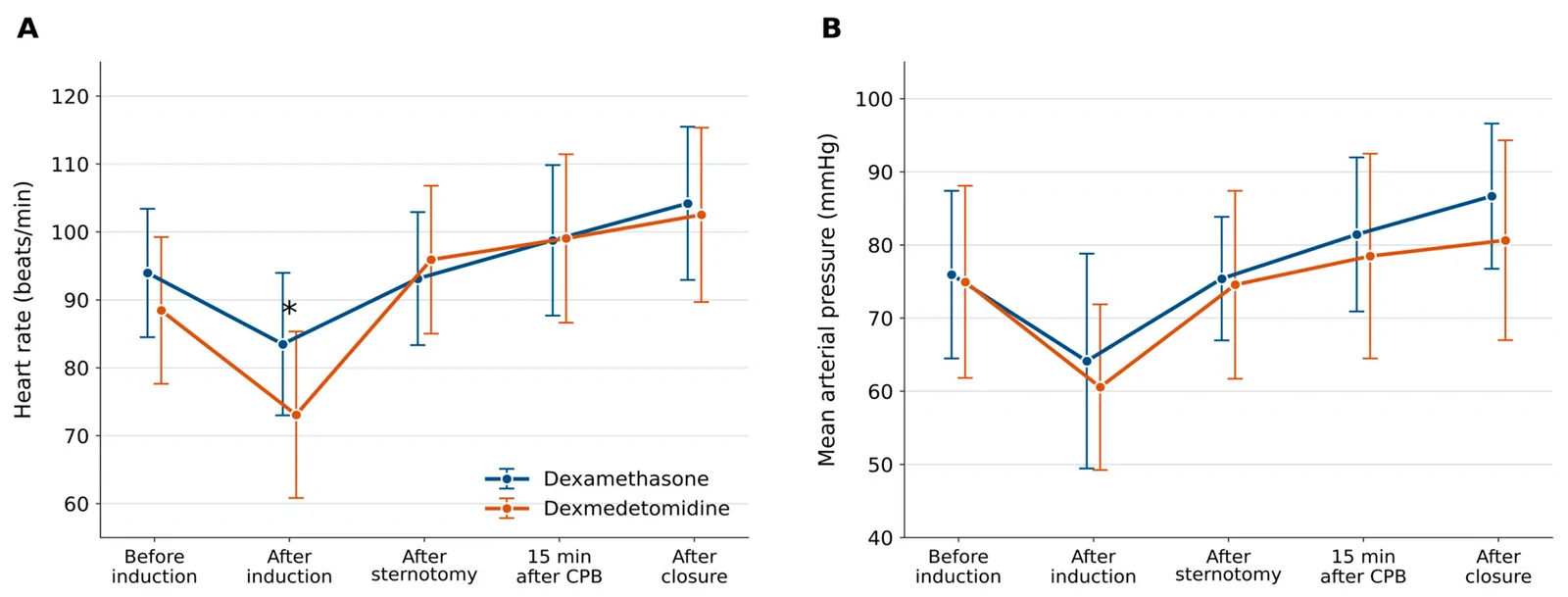
Intraoperative hemodynamic parameters in the dexamethasone and dexmedetomidine groups. (**A**) Heart rate and (**B**) mean arterial pressure at five perioperative time points. Data are presented as means with standard deviation error bars. Heart rate was lower in the dexmedetomidine group after induction of anesthesia (*p* = 0.002), with no significant between-group differences at the remaining time points. Mean arterial pressure did not differ significantly between groups at any time point. The asterisk in panel (**A**) denotes the between-group comparison at the corresponding time point. Heart rate and mean arterial pressure were secondary outcomes; the time-point comparisons were not adjusted for multiple comparisons, and the corresponding *p*-values should therefore be interpreted descriptively. Points were horizontally offset to improve visualization of the error bars. Full numerical data are provided in Supplementary Table S2. CPB, cardiopulmonary bypass.

In the pre-specified time-point comparisons, heart rate was lower in the dexmedetomidine group after induction of anesthesia (73.08 ± 12.24 vs. 83.48 ± 10.51 beats/min; mean difference 10.40 beats/min, 95% CI 3.91 to 16.89; *p* = 0.002), with no significant between-group differences at the remaining time points. Mean arterial pressure did not differ significantly between groups at any measured time point (Figure 4); means, mean differences with 95% CIs, and *p*-values for all time points are provided in Supplementary Table S2. In post hoc linear mixed-effects analyses, the heart-rate trajectory differed between groups (group-by-time interaction *F*(4, 192) = 25.06, *p* < 0.001), consistent with the lower heart rate after induction in the dexmedetomidine group, whereas the overall group effect was not significant (estimated marginal mean difference 2.9 beats/min, 95% CI −3.2 to 9.0; *p* = 0.342). For mean arterial pressure, neither the group effect (estimated marginal mean difference 2.9 mmHg, 95% CI −2.9 to 8.6; *p* = 0.318) nor the group-by-time interaction (*p* = 0.413) was significant. Heart rate and mean arterial pressure were secondary outcomes, and these comparisons were not adjusted for multiple comparisons.

#### 3.3.3. Recovery and Safety Outcomes

Time to extubation did not differ significantly between groups (median 2 h [IQR 1–5] in the dexmedetomidine group vs. 1 h [IQR 1–6] in the dexamethasone group; Hodges–Lehmann difference 0 h, 95% CI −1 to 0; *p* = 0.204). ICU length of stay, which was recorded in days, was distributed toward shorter stays in the dexmedetomidine group (median 2 days [IQR 1–2] vs. 2 days [IQR 2–3]; Hodges–Lehmann difference 1 day, 95% CI 0 to 1; nominal *p* = 0.007) (Table 3). Because ICU length of stay was an exploratory outcome for which the study was not powered, was not adjusted for multiple comparisons, and was recorded with whole-day resolution, this finding is hypothesis-generating and should be interpreted with caution.

No postoperative complications were recorded in either group. Specifically, there were no cases of reintubation, postoperative nausea and vomiting, respiratory depression, pruritus, or neurological deficit. No episodes of bradycardia or hypotension meeting the pre-specified intervention thresholds occurred, and no participant required additional vasoactive support attributable to the study drug.

## 4. Discussion

In this exploratory randomized trial, intraoperative dexmedetomidine was associated with lower early postoperative IL-6 and IL-8 concentrations and lower opioid consumption than dexamethasone. For the primary IL-6 endpoint, the difference was robust at 12 h but imprecise post-CPB, where it would not withstand post hoc correction for the two primary comparisons. These findings are consistent with previous pediatric randomized trials and systematic reviews showing that dexmedetomidine can attenuate inflammatory mediators, including IL-6, during pediatric cardiac surgery with CPB [20,21,22]. However, the results should be interpreted cautiously in view of the exploratory design, small sample size, biomarker-focused outcomes, and the comparison of two institutional regimens that were not equipotent anti-inflammatory doses.

The lower inflammatory biomarker concentrations observed with dexmedetomidine should not be interpreted as evidence of improved clinical recovery. Large randomized trials of anti-inflammatory strategies in pediatric cardiac surgery, including the STRESS trial of methylprednisolone in 1263 infants, have demonstrated biochemical effects without corresponding improvements in clinically important outcomes [15]. Similarly, pediatric meta-analyses have shown that corticosteroids may suppress inflammation while having inconsistent effects on mortality and other clinical endpoints [13,14,30]. The same caution applies to dexmedetomidine; although pediatric meta-analytic evidence supports reductions in IL-6, evidence for sustained patient-centered benefit remains limited [21]. Therefore, the present biomarker findings are best interpreted as pharmacodynamic signals rather than evidence of clinical superiority.

The anti-inflammatory effects of dexmedetomidine are also not uniform across the literature. In a 2025 randomized trial of 100 adults undergoing coronary artery bypass grafting, intraoperative dexmedetomidine at 0.5 µg/kg/h without a loading dose did not attenuate postoperative increases in IL-6, C-reactive protein, white blood cell count, or fibrinogen [24]. Differences in population, dosing strategy, and biomarker sampling may partly explain the discrepancy between that study and the present findings. These observations suggest that the magnitude of the inflammatory response to dexmedetomidine may be dose- and context-dependent and should not be generalized beyond the regimen and population studied here.

The mechanisms underlying the observed biomarker differences remain uncertain. Dexmedetomidine may directly modulate inflammatory signaling [31], but lower cytokine concentrations may also result indirectly from sympatholysis and antinociception, with attenuation of the perioperative stress response [17,19,25]. Experimental studies have implicated inhibition of nuclear factor-κB signaling, reduced high-mobility group box 1 expression and secretion, and suppression of tumor necrosis factor-α, IL-6, and IL-8 production [31,32]. Although the lower IL-8 concentrations observed in this study are compatible with these mechanisms, nuclear factor-κB activity and high-mobility group box 1 were not measured, and a direct immunomodulatory mechanism cannot be established. Serum cortisol also did not differ between groups, but a single postoperative measurement provides only a limited assessment of the overall surgical stress response.

Importantly, associations between inflammatory biomarkers and postoperative outcomes should not be interpreted as causal. IL-6 and IL-8 have been associated with acute kidney injury, prolonged ventilation, and low cardiac output syndrome following pediatric cardiac surgery [9,10]. However, the present study was not designed to determine whether reducing these biomarkers improves renal, neurological, respiratory, or other patient-centered outcomes.

### 4.1. Analgesia and Opioid Sparing

The analgesic findings represent the most clinically tangible signal in this trial. Dexmedetomidine was associated with 42% lower recorded intraoperative fentanyl consumption (a relative reduction that is probably overstated because intraoperative documentation was incomplete; Section 4.5) and 49% lower cumulative fentanyl consumption during the first 24 postoperative hours, and with a time to first rescue analgesia that was approximately 2.8 h longer. These findings are consistent with the known analgesic and sympatholytic properties of dexmedetomidine [17].

Opioid-sparing strategies are particularly relevant in pediatric perioperative care because opioid exposure may contribute to respiratory depression and delayed recovery. However, opioid consumption is a surrogate process outcome that depends partly on clinical decision-making, and the present trial was not powered to determine whether the lower fentanyl consumption translated into shorter duration of ventilation, shorter ICU length of stay, or other clinical benefits. Time to extubation did not differ between groups, and the shorter ICU length of stay observed in an exploratory, unadjusted analysis was recorded only in whole days and should not be regarded as evidence of benefit. The clinical significance of the observed opioid difference therefore requires confirmation in larger trials with patient-centered endpoints, such as duration of mechanical ventilation, respiratory complications, and quality of recovery. In addition, FLACC scores increased progressively over the first 24 postoperative hours in both groups despite rescue analgesia. Therefore, lower opioid consumption should not be interpreted as evidence that either regimen provided optimal analgesia throughout the postoperative period.

Several features of the trial may have influenced the opioid findings independently of any pharmacological effect. First, the intraoperative anesthesia team was not blinded to allocation, and supplemental fentanyl was administered partly according to clinical judgment and predefined hemodynamic triggers. Because dexmedetomidine lowers heart rate and attenuates sympathetic responses [17], heart-rate-based triggers would be expected to occur less often in the dexmedetomidine group; for this reason, supplemental fentanyl decisions in that group were guided primarily by mean arterial pressure and the surgical context (Section 2.4). This asymmetry, together with knowledge of the allocated treatment, could have introduced performance bias in fentanyl administration and measurement bias in the intraoperative fentanyl endpoint, so the intraoperative opioid-sparing effect may partly reflect pharmacological masking of sympathetic signs or differences in clinician behavior rather than a true reduction in analgesic requirement. In a post hoc regression adjusting for heart rate and mean arterial pressure after induction, the between-group difference persisted (adjusted mean difference 63.8 µg, 95% CI 38.8 to 88.8); however, these hemodynamic values may themselves have been affected by the study drug (heart rate after induction was lower in the dexmedetomidine group) and were recorded at a single time point, so this analysis cannot exclude performance bias. In addition, intraoperative fentanyl documentation was incomplete (Section 4.5), which further limits the reliability of the intraoperative comparison. Postoperative fentanyl consumption, FLACC scores, and time to first rescue analgesia were recorded by personnel blinded to allocation, which reduces but does not eliminate this concern, because residual sedation after dexmedetomidine could lower behavioral pain scores and influence analgesic decisions. Second, fentanyl consumption was analyzed in absolute micrograms rather than per kilogram. Mean body weight was numerically higher in the dexmedetomidine group, which would not be expected to explain lower absolute consumption in that group; nevertheless, given the wide age and weight range, the observed opioid difference may partly reflect differences in body size or dosing requirements.

### 4.2. Population Heterogeneity

The study population included a broad spectrum of congenital cardiac lesions and procedures, ranging from relatively simple defect repairs to single-ventricle palliation. This reflects routine pediatric cardiac surgical practice but also introduces heterogeneity in baseline physiology, operative complexity, CPB exposure, and inflammatory burden. The magnitude of CPB-associated inflammation is known to vary according to lesion complexity [8].

Previous pediatric data have demonstrated a greater postoperative increase in the soluble receptor for advanced glycation end products, a marker of inflammation and lung injury, in children undergoing single-ventricle palliation compared with biventricular repair [33]. In the present trial, single-ventricle procedures were numerically imbalanced between the two groups (four in the dexamethasone group and seven in the dexmedetomidine group), and isolated ventricular septal defect closures, which are relatively simple repairs, were more frequent in the dexamethasone group (6 vs. 1; Table 1). Because procedure type may influence both the magnitude of the inflammatory response and postoperative analgesic requirements, these imbalances are directly relevant to the interpretation of the primary IL-6 findings, the secondary IL-8 findings, and the opioid findings. If single-ventricle palliation and other more complex procedures are associated with a greater inflammatory response than simple repairs [8,33], these two imbalances, which were the largest in Table 1, would tend to work against, rather than in favor of, lower biomarker concentrations in the dexmedetomidine group, although smaller imbalances in other procedures, such as valve procedures and truncus arteriosus repair, ran in the opposite direction. In addition, CPB and aortic cross-clamp times, which reflect the duration of exposure to the extracorporeal circuit and of myocardial ischemia, were similar between groups (Table 1), making it unlikely that differences in CPB exposure account for the biomarker findings. However, single-ventricle procedures also differ from biventricular repairs in physiology, operative course, and postoperative management, so the net direction and magnitude of any bias on the biomarker and opioid outcomes cannot be determined from these data, and the observed differences cannot be attributed to the study drugs alone. Adjustment or stratification by surgical complexity was not undertaken because the small numbers of individual procedures, including single-ventricle procedures (4 vs. 7), and the wide range of lesions would not support stable adjusted or stratified estimates; the post hoc baseline-adjusted biomarker analyses accounted for baseline concentrations but not for differences in operative procedures. This heterogeneity may also have contributed to within-group variability in IL-6 and IL-8 concentrations. Future adequately powered studies should stratify randomization by surgical complexity, for example using the Society of Thoracic Surgeons–European Association for Cardio-Thoracic Surgery (STS-EACTS) Congenital Heart Surgery Mortality Categories [34].

### 4.3. Dosing Regimens and Pharmacokinetic Considerations

The lack of pharmacological equivalence between the two regimens is central to the interpretation of this trial (Section 2.3). Because a glucocorticoid and an α2-adrenergic agonist act through different mechanisms, no dose of one can be regarded as equivalent to a given dose of the other; the between-group differences therefore reflect the two regimens as administered and do not indicate which agent has greater intrinsic anti-inflammatory potency. The results apply only to the regimens studied: dexamethasone 1 mg/kg (maximum 12 mg) at three perioperative time points, and dexmedetomidine 0.5 µg/kg followed by 0.1 µg/kg/h until skin closure, which corresponded to the lower of two dose regimens evaluated in a previous trial in children aged 6–12 years undergoing septal defect repair [20]; whether this regimen is optimal across the wider age range and lesion spectrum of the present trial is unknown. A different dexamethasone dose or schedule, or a higher dose or longer duration of dexmedetomidine, might have produced different biomarker, analgesic, and hemodynamic results, and the present data cannot define dose–response relationships for either agent. Dose-ranging studies, ideally including a placebo arm, would be required to establish the contribution of each agent and dose.

The persistence of lower inflammatory biomarker concentrations after discontinuation of dexmedetomidine raises the possibility that the timing of administration during peak CPB-related inflammatory stress may be important. The biological effects of pathway modulation may outlast measurable drug exposure. This differs from dexamethasone, which has a longer duration of action and was administered at additional perioperative time points. Accordingly, the timing and pattern of drug exposure may influence the observed inflammatory response. This interpretation remains mechanistic and hypothesis-generating because the trial was not designed to investigate pharmacokinetic–pharmacodynamic relationships.

### 4.4. Tolerability

Dexmedetomidine was associated with the expected lower heart rate following induction of anesthesia, without significant hypotension or increased vasoactive support. This is consistent with previous evidence demonstrating that dexmedetomidine can reduce heart rate while maintaining overall hemodynamic stability in children undergoing cardiac surgery [35].

No pre-specified adverse events occurred in either group. However, the small sample size limits conclusions regarding safety, and the absence of observed adverse events should not be interpreted as evidence that clinically important adverse effects are absent. In addition, perioperative hyperglycemia and insulin use, which are recognized adverse effects of corticosteroids [14,15], were not systematically recorded, so the comparative metabolic safety of the two regimens could not be assessed. Larger studies are required to characterize comparative safety more reliably.

### 4.5. Limitations

This study has several limitations. First, it was a single-center exploratory randomized trial with a small sample size, which limits statistical precision and generalizability. Although standardized perioperative protocols supported internal consistency, the intraoperative anesthesia team could not be fully blinded to treatment allocation, which may have introduced performance bias in intraoperative fentanyl administration (Section 4.1).

Second, apart from the post hoc Bonferroni-adjusted threshold applied to the two primary IL-6 comparisons, multiple biomarker measurements and numerous secondary and exploratory outcomes, including IL-8 at two time points, FLACC scores at six time points, opioid consumption, and ICU length of stay, were assessed without adjustment for multiple comparisons. The pre-specified analyses compared repeated measurements separately at individual time points; the longitudinal mixed-effects analyses were added post hoc as sensitivity analyses, and a longitudinal ordinal model for FLACC could not be estimated. Therefore, secondary and exploratory *p*-values are nominal and should be interpreted descriptively, and the possibility of type I error cannot be excluded.

Third, fentanyl consumption was analyzed in absolute units rather than normalized to body weight. Furthermore, we noted a discrepancy in the intraoperative fentanyl data extracted from the anesthesia records: the recorded totals were lower than would be expected from the standardized protocol, which included a 5 µg/kg induction dose followed by a weight-based infusion. For example, the mean recorded intraoperative consumption in the dexmedetomidine group (83.0 µg) was lower than the protocol induction dose alone at that group’s mean body weight (approximately 100 µg). This suggests that some components of intraoperative fentanyl administration (such as the induction dose or the infusion totals) were incompletely documented or not captured during data extraction; the recorded values therefore underestimate total intraoperative exposure and do not permit a precise weight-normalized analysis. The impact on the opioid-sparing estimate depends on whether the undocumented amounts differed between groups. If they were similar, as would be expected for protocol-specified, weight-based components in groups of comparable body weight, the absolute between-group difference would be largely preserved, but the relative reduction would be overstated because it is calculated from underestimated totals; for instance, if the undocumented component were the protocol induction dose, adding it at each group’s mean body weight would leave an absolute difference of approximately 51 µg but would reduce the relative difference from 42% to approximately 22%. Because the intraoperative anesthesia team was not blinded, we cannot exclude that incomplete documentation differed between groups, in which case the direction and magnitude of the resulting bias cannot be determined. The intraoperative fentanyl difference should therefore be interpreted with caution and only as an approximate between-group comparison, and the 42% figure should not be regarded as a precise estimate of the intraoperative opioid-sparing effect. Postoperative fentanyl consumption and time to first rescue analgesia, which were recorded by personnel blinded to allocation, differed in the same direction and are less susceptible to this documentation issue, although they remain subject to the limitations discussed in Section 4.1. Given the broad pediatric age and weight range, the reported absolute opioid differences may also partly reflect differences in body size or dosing requirements. Future trials must ensure meticulous, prospective, component-specific opioid recording.

Fourth, biomarker concentrations may be influenced by pre-analytical factors such as the interval between blood collection and centrifugation, centrifugation conditions, storage duration, and freeze–thaw cycles. Although specimens were processed according to a standardized protocol, these factors were not individually recorded for each sample. In addition, some IL-6 concentrations at baseline and post-CPB were below the lower limit of detection and are therefore imprecise (Section 2.5); moreover, the laboratory records did not document which samples were below the lower limit of detection or required reassay after dilution.

Fifth, follow-up was limited to the early postoperative period. The study did not evaluate longer-term analgesic requirements, neurodevelopmental outcomes, renal outcomes, or other patient-centered measures. Future studies should extend postoperative follow-up and incorporate clinically meaningful outcomes.

Sixth, the two treatment regimens were not pharmacologically dose-equivalent and differed in the timing and duration of drug exposure (Section 2.3 and Section 4.3). Each reflected the dose and schedule routinely used at the study institution for its respective clinical indication. Therefore, the findings compare two clinical regimens rather than equivalent or maximal anti-inflammatory doses of the two agents. Moreover, perioperative glucose concentrations, hyperglycemic episodes, and insulin use were not systematically recorded, which precluded comparison of the metabolic adverse effects associated with corticosteroids.

Seventh, time to first rescue analgesia was measured according to scheduled clinical assessment rather than continuous observation. The observed between-group difference should therefore be interpreted as an approximate measure rather than a highly precise timing estimate.

Eighth, the inflammatory biomarker panel was limited to IL-6 and IL-8. Other relevant mediators, including tumor necrosis factor-α, IL-1β, IL-10, and nuclear factor-κB activity, were not measured. In addition, FLACC is an ordinal scale; inferential comparisons were therefore based on rank-based methods, and means and standard deviations are presented descriptively only.

Ninth, exclusion of patients undergoing emergency surgery and those with pre-existing renal or hepatic dysfunction limits applicability to higher-risk pediatric cardiac surgical populations.

Tenth, baseline IL-6 concentrations were imbalanced between groups despite randomization. This likely reflects chance imbalance in a small sample with a markedly skewed biomarker distribution, although a contribution of differences in case mix cannot be excluded (Section 4.2). Although a baseline-adjusted analysis on the log-transformed scale yielded consistent results, it was performed post hoc and should be interpreted as supportive rather than confirmatory.

Finally, the cohort included heterogeneous cardiac lesions and a numerical imbalance in individual surgical procedures, including single-ventricle palliation, which may have influenced the biomarker and opioid outcomes in either direction. The small sample size precluded meaningful adjustment, stratification, or subgroup analyses according to age, lesion complexity, or procedure (Section 4.2). ICU length of stay and time to extubation were exploratory outcomes, and the study was not powered to detect between-group differences in these endpoints; ICU length of stay was also recorded only in whole days.

### 4.6. Interpretation and Implications

The findings of this exploratory trial are primarily pharmacodynamic and analgesic and should be considered hypothesis-generating. This applies particularly to the secondary and exploratory outcomes, including IL-8, FLACC scores, opioid consumption, time to first rescue analgesia, and ICU length of stay, which were not adjusted for multiple comparisons and whose nominal *p*-values should not be interpreted as confirmatory. Three levels of evidence should be distinguished: differences in inflammatory biomarkers, differences in opioid consumption and pain scores, and patient-centered clinical outcomes. Differences were observed at the first two levels, whereas no patient-centered clinical benefit was demonstrated. The findings do not establish therapeutic superiority of dexmedetomidine over dexamethasone and do not support changes to current clinical practice on their own. Both agents retain established roles in pediatric cardiac anesthesia, and treatment selection should continue to consider patient-specific factors, clinical context, and their differing adverse-effect profiles.

The present findings nevertheless provide a rationale for a larger comparative trial. Future studies should be adequately powered, stratify randomization by surgical complexity, blind intraoperative analgesic management where feasible, use longitudinal statistical models for repeated biomarker measurements, incorporate weight-normalized opioid outcomes, systematically record glycemic outcomes, extend follow-up to patient-centered clinical endpoints, and pre-specify a single principal primary endpoint.

## 5. Conclusions

In this exploratory randomized controlled trial of children undergoing congenital heart surgery with CPB, which compared two institutionally used regimens that were not equipotent anti-inflammatory doses, dexmedetomidine was associated with lower early postoperative IL-6 and IL-8 concentrations and lower opioid consumption compared with dexamethasone. These findings, particularly those for the secondary and exploratory outcomes (IL-8, FLACC scores, opioid consumption, and ICU length of stay), which were not adjusted for multiple comparisons, should be interpreted as hypothesis-generating pharmacodynamic and analgesic signals rather than evidence of therapeutic superiority or patient-centered clinical benefit. The observed biomarker differences may reflect both direct immunomodulatory effects and indirect effects related to sympatholysis and antinociception. Larger, adequately powered multicenter trials with stratification by surgical complexity and longer-term, patient-centered outcomes are needed to determine whether these biomarker and analgesic effects translate into meaningful clinical benefit.

## Figures and Tables

**Table 1:** Demographic, clinical, and perioperative characteristics of the two study groups.

Characteristic	Dexamethasone (*n* = 25)	Dexmedetomidine (*n* = 25)	Difference ^a^	95% CI
Age, years, mean ± SD	4.80 ± 3.04	5.50 ± 3.60	−0.70	−2.59 to 1.19
Male sex, *n* (%)	13 (52)	10 (40)	—	—
Weight, kg, mean ± SD	18.28 ± 7.92	20.06 ± 8.62	−1.78	−6.49 to 2.93
Aortic cross-clamp time, min, mean ± SD	50.80 ± 12.64	48.32 ± 19.78	2.48	−6.96 to 11.92
CPB time, min, mean ± SD	63.00 ± 13.38	64.32 ± 22.35	−1.32	−11.79 to 9.15
Single-ventricle palliation, *n*	4	7	—	—
Biventricular repair, *n*	21	18	—	—
Diagnosis/procedure, *n*				
Septal defect closure	9	8	—	—
Ventricular septal defect closure	6	1	—	—
Atrial and ventricular septal defect closure	2	4	—	—
Atrial septal defect closure	1	1	—	—
Partial atrioventricular canal repair	0	2	—	—
Conotruncal repair	8	8	—	—
Tetralogy of Fallot repair	5	5	—	—
Transposition of the great arteries (arterial switch)	2	3	—	—
Truncus arteriosus, total repair	1	0	—	—
Valve repair or replacement	4	2	—	—
Aortic valve repair	2	2	—	—
Pulmonary valve repair	1	0	—	—
Mitral valve replacement	1	0	—	—
Single-ventricle palliation	4	7	—	—
Glenn operation	2	2	—	—
Fontan operation	2	4	—	—
Blalock–Taussig shunt	0	1	—	—

^a^ Difference in means (dexamethasone minus dexmedetomidine) with 95% CI calculated using the pooled-variance *t*-distribution; in accordance with CONSORT guidance, baseline characteristics were not subjected to significance testing. CPB, cardiopulmonary bypass; CI, confidence interval; SD, standard deviation.

**Table 2:** Serum IL-6 (primary outcome) and IL-8 (secondary outcome) concentrations by group and time point.

Biomarker/Time Point	Dexamethasone (*n* = 25)	Dexmedetomidine (*n* = 25)	Hodges–Lehmann Median Difference (95% CI) ^a^	Effect Size *r* ^b^	*p*-Value
IL-6, baseline, pg/mL	53 (2–108)	12 (2–370)	14 (−2 to 49)	—	Not tested ^c^
IL-6, post-CPB, pg/mL	77 (12–340)	46 (2–288)	34 (0 to 112)	0.28	0.045 *
IL-6, 12 h postoperatively, pg/mL	178 (45–360)	50 (11–290)	86 (59 to 144)	0.68	<0.001 *
IL-8, baseline, pg/mL	448 (111–717)	412 (94–726)	63 (−38 to 185)	—	Not tested ^c^
IL-8, post-CPB, pg/mL	381 (106–797)	159 (50–543)	160 (76 to 267)	0.45	0.001 *
IL-8, 12 h postoperatively, pg/mL	311 (93–841)	120 (20–755)	159 (37 to 244)	0.44	0.002 *

Data are median (minimum–maximum). Between-group comparisons were made with the Mann–Whitney *U* test. ^a^ Hodges–Lehmann estimate of the median difference (dexamethasone minus dexmedetomidine) with a distribution-free 95% CI, in pg/mL; this location-shift estimate corresponds to the rank-based test performed. ^b^ Effect size *r*, calculated as *z*/√*N* (*N* = 50), where 0.1, 0.3, and 0.5 correspond conventionally to small, medium, and large effects; a positive value denotes lower concentrations in the dexmedetomidine group. ^c^ Not tested: in accordance with CONSORT guidance, baseline concentrations were not subjected to significance testing; they are presented descriptively, and the baseline imbalance in IL-6 was addressed by adjustment in the post hoc longitudinal sensitivity analysis. * *p* < 0.05. For IL-6 (primary outcome), the post hoc Bonferroni-adjusted threshold for the two postoperative comparisons is 0.025, which is met at 12 h but not post-CPB; for IL-8 (secondary outcome), *p*-values are nominal and unadjusted for multiplicity. In the post hoc baseline-adjusted linear mixed-effects model of log_10_-transformed concentrations, the ratio of adjusted geometric means (dexmedetomidine/dexamethasone) across the two postoperative time points was 0.35 (95% CI 0.22 to 0.58; *p* < 0.001) for IL-6 and 0.50 (95% CI 0.36 to 0.71; *p* < 0.001) for IL-8. IL-6 values below the lower limit of detection were retained as recorded (Section 2.5). CPB, cardiopulmonary bypass; CI, confidence interval; IL, interleukin.

**Table 3:** Secondary, exploratory, and safety outcomes by group.

Outcome	Dexamethasone (*n* = 25)	Dexmedetomidine (*n* = 25)	Effect Estimate (95% CI) ^a^	Test	*p*-Value
Intraoperative fentanyl, µg, mean ± SD	142.6 ± 51.7	83.0 ± 30.7	59.6 (35.4 to 83.8)	*t* = 4.95	<0.001 *
Postoperative 24-h fentanyl, µg, mean ± SD	105.4 ± 32.8	53.8 ± 17.4	51.6 (36.7 to 66.5)	*t* = 6.95	<0.001 *
Time to first administration of rescue analgesia, h, mean ± SD	1.84 ± 0.23	4.68 ± 0.73	−2.84 (−3.15 to −2.53)	*t* = 18.39	<0.001 *
FLACC score, median [IQR]					
1 h	2 [2–2]	2 [1–2]	0 (0 to 0)	*z* = 1.90	0.057
2 h	5 [4–5]	2 [2–2]	3 (3 to 3)	*z* = 6.57	<0.001 *
6 h	5 [4.5–5]	4 [4–5]	1 (0 to 1)	*z* = 2.47	0.014 *
12 h	6 [6–6]	4 [4–5]	1 (1 to 2)	*z* = 5.33	<0.001 *
18 h	6 [6–7]	5 [5–6]	1 (0 to 1)	*z* = 3.45	0.001 *
24 h	7 [7–7]	6 [5–6]	1 (1 to 2)	*z* = 5.36	<0.001 *
Serum cortisol, baseline, µg/dL, mean ± SD	13.56 ± 2.25	12.76 ± 2.05	0.80 (−0.43 to 2.03)	—	Not tested ^b^
Serum cortisol, 1 h postoperatively, µg/dL, mean ± SD	32.24 ± 2.70	31.72 ± 1.59	0.52 (−0.74 to 1.78)	*t* = 0.83	0.411
Time to extubation, h, median [IQR]	1 [1–6]	2 [1–5]	0 (−1 to 0)	*z* = 1.27	0.204
ICU length of stay, days, median [IQR]	2 [2–3]	2 [1–2]	1 (0 to 1)	*z* = 2.69	0.007 *
Postoperative complications, *n* (%)					
Reintubation	0 (0)	0 (0)	—	—	—
Postoperative nausea and vomiting	0 (0)	0 (0)	—	—	—
Respiratory depression	0 (0)	0 (0)	—	—	—
Pruritus	0 (0)	0 (0)	—	—	—
Neurological deficit	0 (0)	0 (0)	—	—	—

Continuous variables are mean ± SD or median [IQR], as indicated. ^a^ For variables summarized as mean ± SD, the effect estimate is the difference in means (dexamethasone minus dexmedetomidine) with a 95% CI based on the pooled-variance *t*-distribution (independent-samples *t*-test); for variables summarized as median [IQR], it is the Hodges–Lehmann median difference (dexamethasone minus dexmedetomidine) with a distribution-free 95% CI, and the *p*-value is from the Mann–Whitney *U* test. Intraoperative fentanyl values are as recorded in the anesthesia records, which were incompletely documented (Section 4.5). Because FLACC scores, time to extubation, and ICU length of stay take integer values with many ties, the Hodges–Lehmann confidence limits may coincide with the point estimate or with zero. ^b^ Not tested: in accordance with CONSORT guidance, baseline cortisol was not subjected to significance testing. * Nominal *p* < 0.05; secondary and exploratory outcomes were not adjusted for multiple comparisons. Test statistics are shown as absolute values. FLACC was assessed on a 0–10 scale. ICU length of stay was recorded in days. CI, confidence interval; FLACC, Face, Legs, Activity, Cry, Consolability; ICU, intensive care unit; IQR, interquartile range; SD, standard deviation.

## Data Availability

The datasets generated and analyzed during the current study are not publicly available because the informed consent obtained from participants did not include permission for public data deposition. The data are available from the corresponding authors upon reasonable request and subject to institutional approval. During the preparation of this manuscript, the authors used AI-assisted tools for language editing and reference formatting. The authors subsequently reviewed and edited the output and take full responsibility for the content of the manuscript. No generative AI tools were used to generate, analyze, or interpret the study data.

## Supplementary Materials

The following supporting information can be downloaded at: https://www.mdpi.com/article/10.3390/healthcare14203468/s1, Table S1, Completed CONSORT 2025 checklist; Table S2, Full numerical data for heart rate, mean arterial pressure, and FLACC pain scores at each measured time point.
