# Supplementary material for: Dexmedetomidine Versus Dexamethasone in Pediatric Cardiac Surgery with Cardiopulmonary Bypass: An Exploratory Randomized Controlled Trial of Inflammatory Biomarkers and Opioid Consumption
Source: Healthcare (Basel). 2026 Oct 15;14(20):3468. doi: 10.3390/healthcare14203468 (PMC13704332; doi:10.3390/healthcare14203468)
Supplement: Supplementary file 1 [file healthcare-14-03468-s001.zip › healthcare-4573573-supplementary.pdf]

Supplementary Materials

Table S1. Completed CONSORT 2025 checklist.

Section/topic	No.	CONSORT 2025 checklist item	Where reported in the revised manuscript
**Title and abstract**			
Title and structured abstract	1a	Identification as a randomised trial	Title ("An Exploratory Randomized Controlled Trial")
	1b	Structured summary of the trial design, methods, results, and conclusions	Abstract (Background/Objectives, Methods, Results, Conclusions, and Trial Registration)
**Open science**			
Trial registration	2	Name of trial registry, identifying number (with URL) and date of registration	Abstract (Trial Registration); Section 2.1; Institutional Review Board Statement: Pan African Clinical Trials Registry, PACTR202406581259613, registered 3 June 2024, before enrollment of the first participant in July 2024. The registry URL is not stated in the manuscript (registry website: https://pactr.samrc.ac.za)
Protocol and statistical analysis plan	3	Where the trial protocol and statistical analysis plan can be accessed	No published protocol or separate statistical analysis plan is cited. The registered trial details (design, eligibility criteria, interventions, and outcomes) are publicly accessible in the trial registry record (PACTR202406581259613; item 2). Protocol approval is reported in Section 2.1 and the Institutional Review Board Statement
Data sharing	4	Where and how the individual de-identified participant data (including data dictionary), statistical code, and any other materials can be accessed	Data Availability Statement (data available from the corresponding authors on reasonable request, subject to institutional approval; not publicly deposited because consent did not cover public deposition). Access to statistical code is not stated
Funding and conflicts of interest	5a	Sources of funding and other support (eg, supply of drugs), and role of funders in the design, conduct, analysis, and reporting of the trial	Funding statement (no external funding)
	5b	Financial and other conflicts of interest of the manuscript authors	Conflicts of Interest statement (none declared)
**Introduction**			
Background and rationale	6	Scientific background and rationale	Section 1 (Introduction)
Objectives	7	Specific objectives related to benefits and harms	Section 1, final paragraph (objective and hypothesis); safety outcomes are defined in Section 2.6
**Methods**			
Patient and public involvement	8	Details of patient or public involvement in the design, conduct, and reporting of the trial	Not reported
Trial design	9	Description of trial design including type of trial (eg, parallel group, crossover), allocation ratio, and framework (eg, superiority, equivalence, non-inferiority, exploratory)	Section 2.1 (prospective, randomized, controlled trial with an exploratory framework; reasons it is not classified as a pilot or feasibility trial); Section 2.2 (1:1 allocation to the dexamethasone or dexmedetomidine group)

Section/topic	No.	CONSORT 2025 checklist item	Where reported in the revised manuscript
Changes to trial protocol	10	Important changes to the trial after it commenced including any outcomes or analyses that were not prespecified, with reason	Section 2.6 (IL-8, pre-specified as a co-primary biomarker outcome, is reported as a secondary outcome because the study was not powered for it); Section 2.7 (analyses that were not pre-specified are identified as post hoc, with reasons)
Trial setting	11	Settings (eg, community, hospital) and locations (eg, countries, sites) where the trial was conducted	Section 2.1 (approving institution: Faculty of Medicine, Mansoura University, Mansoura, Egypt); Section 2.4 (intraoperative care and postoperative ICU); Section 4.5 (single-center trial)
Eligibility criteria	12a	Eligibility criteria for participants	Section 2.1, second paragraph
	12b	If applicable, eligibility criteria for sites and for individuals delivering the interventions (eg, surgeons, physiotherapists)	Not applicable (single-center drug trial; study drugs administered by the intraoperative anesthesia team, Section 2.2)
Intervention and comparator	13	Intervention and comparator with sufficient details to allow replication. If relevant, where additional materials describing the intervention and comparator (eg, intervention manual) can be accessed	Section 2.3 (2.3.1, dexamethasone regimen; 2.3.2, dexmedetomidine regimen; matched placebo; rationale and non-equipotency of the two regimens); Section 2.4 (standardized anesthetic, surgical, CPB, and postoperative analgesic management)
Outcomes	14	Prespecified primary and secondary outcomes, including the specific measurement variable (eg, systolic blood pressure), analysis metric (eg, change from baseline, final value, time to event), method of aggregation (eg, median, proportion), and time point for each outcome	Section 2.6 (primary outcome: IL-6 post-CPB and 12 h postoperatively; secondary and exploratory outcomes); Section 2.5 (sampling time points and assays); Section 2.4 (FLACC time points, rescue analgesia, and time to first rescue analgesia); Section 2.7 (analysis metrics and summary measures)
Harms	15	How harms were defined and assessed (eg, systematically, non-systematically)	Section 2.6, final paragraph (definitions of intraoperative bradycardia and hypotension; postoperative complications recorded in all participants); Section 2.3.1 (glucose concentrations, hyperglycemic episodes, and insulin use not systematically recorded)
Sample size	16a	How sample size was determined, including all assumptions supporting the sample size calculation	Section 2.7, first paragraph
	16b	Explanation of any interim analyses and stopping guidelines	Not applicable (no interim analyses or stopping guidelines are described; the planned sample of 50 participants was enrolled, Sections 2.7 and 3.1)
Sequence generation	17a	Who generated the random allocation sequence and the method used	Section 2.2 (computer-generated sequence prepared by an investigator not involved in patient care, outcome assessment, or data collection)
	17b	Type of randomisation and details of any restriction (eg, stratification, blocking and block size)	Section 2.2 (1:1 allocation in fixed permuted blocks of four; the potential predictability of fixed blocks is acknowledged)

Section/topic	No.	CONSORT 2025 checklist item	Where reported in the revised manuscript
Allocation concealment mechanism	18	Mechanism used to implement the random allocation sequence (eg, central computer/telephone; sequentially numbered, opaque, sealed containers), describing any steps to conceal the sequence until interventions were assigned	Section 2.2 (sequentially numbered, opaque, sealed envelopes opened only after induction of anesthesia)
Implementation	19	Whether the personnel who enrolled and those who assigned participants to the interventions had access to the random allocation sequence	Section 2.2 (sequence and envelopes prepared by an investigator not involved in care; enrollment by the attending pediatric anesthesiologist; allocation by a member of the intraoperative anesthesia team after envelope opening)
Blinding	20a	Who was blinded after assignment to interventions (eg, participants, care providers, outcome assessors, data analysts)	Section 2.2, third paragraph (intraoperative anesthesia team not fully blinded; postoperative data collectors, outcome assessors, laboratory personnel, and statistical analysts blinded); Sections 2.4 and 2.5
	20b	If blinded, how blinding was achieved and description of the similarity of interventions	Section 2.2, third paragraph, and Sections 2.3.1–2.3.2 (volume- and appearance-matched 0.9% saline placebo given at the time points of the comparator regimen)
Statistical methods	21a	Statistical methods used to compare groups for primary and secondary outcomes, including harms	Section 2.7 (statistical tests, effect estimates, significance thresholds, and handling of multiplicity)
	21b	Definition of who is included in each analysis (eg, all randomised participants), and in which group	Section 3.1 and Figure 1 (all 50 randomized participants analyzed in their allocated groups)
	21c	How missing data were handled in the analysis	No participant was lost to follow-up and all randomized participants were analyzed (Section 3.1; Figure 1); IL-6 values below the lower limit of detection were retained as recorded (Section 2.5); intraoperative fentanyl was analyzed as recorded despite incomplete documentation (Table 3 footnote; Section 4.5)
	21d	Methods for any additional analyses (eg, subgroup and sensitivity analyses), distinguishing prespecified from post hoc	Section 2.7 (baseline-adjusted and longitudinal mixed-effects models, fentanyl regression, attempted ordinal model, and Bonferroni-adjusted threshold, all identified as post hoc sensitivity analyses); no subgroup analyses (Section 4.5)
**Results**			
Participant flow, including flow diagram	22a	For each group, the numbers of participants who were randomly assigned, received intended intervention, and were analysed for the primary outcome	Figure 1 (CONSORT 2025 flow diagram); Section 3.1
	22b	For each group, losses and exclusions after randomisation, together with reasons	Figure 1 and Section 3.1 (no losses or exclusions after randomization)
Recruitment	23a	Dates defining the periods of recruitment and follow-up for outcomes of benefits and harms	Sections 2.1 and 3.1 (July 2024 to March 2025); Sections 2.4–2.6 (outcome assessment time points up to 24 h postoperatively and ICU length of stay); Section 4.5 (early postoperative follow-up only)

Section/topic	No.	CONSORT 2025 checklist item	Where reported in the revised manuscript
	23b	If relevant, why the trial ended or was stopped	Not applicable (the planned sample size was enrolled, Sections 2.7 and 3.1)
Intervention and comparator delivery	24a	Intervention and comparator as they were actually administered (eg, where appropriate, who delivered the intervention/comparator, how participants adhered, whether they were delivered as intended (fidelity))	Section 3.1 and Figure 1 (all participants received the allocated intervention; none discontinued); Section 2.2 (administration by a member of the intraoperative anesthesia team)
	24b	Concomitant care received during the trial for each group	Section 2.4 (standardized anesthetic, CPB, inotropic, and postoperative analgesic care); Table 3 and Section 3.3.1 (fentanyl consumption by group); Section 3.3.3 (vasoactive support); Section 2.3.1 (insulin according to institutional protocol, not systematically recorded)
Baseline data	25	A table showing baseline demographic and clinical characteristics for each group	Table 1 (demographic, clinical, perioperative, and procedural characteristics); baseline IL-6 and IL-8 in Table 2; baseline cortisol in Table 3
Numbers analysed, outcomes and estimation	26	For each primary and secondary outcome, by group: the number of participants included in the analysis; the number of participants with available data at the outcome time point; result for each group, and the estimated effect size and its precision (such as 95% confidence interval); for binary outcomes, presentation of both absolute and relative effect size	Sections 3.2–3.3; Tables 2 and 3 (n = 25 per group; group results with effect estimates and 95% CIs); Figures 2–4; Supplementary Table S2. Binary outcomes (postoperative complications) had no events in either group (Table 3)
Harms	27	All harms or unintended events in each group	Section 3.3.3; Table 3 (postoperative complications by group); Section 4.4
Ancillary analyses	28	Any other analyses performed, including subgroup and sensitivity analyses, distinguishing pre-specified from post hoc	Section 3.2 and Table 2 footnote (post hoc baseline-adjusted mixed-effects models for IL-6 and IL-8); Section 3.3.1 (post hoc fentanyl regression); Section 3.3.2 and Supplementary Table S2 (post hoc mixed-effects models for heart rate and mean arterial pressure)
		**Discussion**	
Interpretation	29	Interpretation consistent with results, balancing benefits and harms, and considering other relevant evidence	Section 4 (introductory paragraphs and Sections 4.1–4.4 and 4.6); Section 5 (Conclusions)
Limitations	30	Trial limitations, addressing sources of potential bias, imprecision, generalisability, and, if relevant, multiplicity of analyses	Section 4.5 (Limitations); Sections 4.1 (performance and measurement bias in opioid outcomes) and 4.2 (population heterogeneity)

Checklist items are reproduced from the CONSORT 2025 statement (Hopewell, S. et al. CONSORT 2025 statement: Updated guideline for reporting randomised trials. *BMJ* **2025**, *389*, e081123; reference 26 of the main text). Locations refer to sections, tables, figures, and back-matter statements of the revised manuscript; section numbers are used instead of page numbers because pagination changes during typesetting. The CONSORT extension for randomised pilot and feasibility trials was not applied because the trial does not meet its definition of a pilot or feasibility trial (Section 2.1). CPB, cardiopulmonary bypass; FLACC, Face, Legs, Activity, Cry, Consolability; ICU, intensive care unit; IL, interleukin.

Table S2. Full numerical data for heart rate, mean arterial pressure, and FLACC pain scores at each measured time point.

Time point	Dexamethasone (n = 25)	Dexmedetomidine (n = 25)	Mean difference (95% CI) [a]	t [b]	p-value
**(A) Heart rate, beats/min, mean ± SD**					
Before induction	93.96 ± 9.45	88.44 ± 10.81	5.52 (−0.25 to 11.29)	1.92	0.061
After induction	83.48 ± 10.51	73.08 ± 12.24	10.40 (3.91 to 16.89)	3.22	0.002 *
After sternotomy	93.12 ± 9.81	95.92 ± 10.88	−2.80 (−8.69 to 3.09)	0.96	0.344
15 min after CPB	98.76 ± 11.07	99.04 ± 12.40	−0.28 (−6.96 to 6.40)	0.08	0.933
After closure	104.20 ± 11.28	102.52 ± 12.85	1.68 (−5.20 to 8.56)	0.49	0.626
**(B) Mean arterial pressure, mmHg, mean ± SD**					
Before induction	75.96 ± 11.46	74.96 ± 13.13	1.00 (−6.01 to 8.01)	0.29	0.775
After induction	64.12 ± 14.70	60.56 ± 11.33	3.56 (−3.90 to 11.02)	0.96	0.342
After sternotomy	75.40 ± 8.47	74.56 ± 12.85	0.84 (−5.35 to 7.03)	0.27	0.786
15 min after CPB	81.44 ± 10.53	78.48 ± 13.99	2.96 (−4.08 to 10.00)	0.85	0.402
After closure	86.68 ± 9.93	80.64 ± 13.67	6.04 (−0.75 to 12.83)	1.79	0.080

Data are mean ± SD; n = 25 per group, with complete data at every time point. These values correspond to those plotted in Figure 4 of the main text. [a] Difference in means (dexamethasone minus dexmedetomidine) with 95% CI based on the pooled-variance t-distribution. [b] Independent-samples Student's t-test (48 degrees of freedom); absolute values of the t statistic are shown, and the direction of each difference is given by the effect estimate. * $p < 0.05$. All time-point comparisons were exploratory and were not adjusted for multiple comparisons. Post hoc linear mixed-effects models (fixed effects: group, time, and group-by-time interaction; random intercept for each participant): heart rate—group $F(1, 48) = 0.92$, $p = 0.342$; time $F(4, 192) = 340.37$, $p < 0.001$; group-by-time interaction $F(4, 192) = 25.06$, $p < 0.001$; estimated marginal mean difference across time points 2.9 beats/min (95% CI −3.2 to 9.0). Mean arterial pressure—group $F(1, 48) = 1.02$, $p = 0.318$; time $F(4, 192) = 56.80$, $p < 0.001$; group-by-time interaction $F(4, 192) = 0.99$, $p = 0.413$; estimated marginal mean difference across time points 2.9 mmHg (95% CI −2.9 to 8.6).

Time after surgery	Dexamethasone (n = 25)		Dexmedetomidine (n = 25)		Hodges–Lehmann median difference (95% CI) [b]	Mann–Whitney U [c]	z [c]	p-value [c]
	Median [IQR]	Mean ± SD [a]	Median [IQR]	Mean ± SD [a]				
**(C) FLACC pain score (0–10 scale)**								
1 h	2 [2–2]	1.92 ± 0.49	2 [1–2]	1.64 ± 0.49	0 (0 to 0)	234.0	1.90	0.057
2 h	5 [4–5]	4.64 ± 0.49	2 [2–2]	1.96 ± 0.20	3 (3 to 3)	0.0	6.57	<0.001 *
6 h	5 [4.5–5]	4.92 ± 0.64	4 [4–5]	4.32 ± 0.90	1 (0 to 1)	194.5	2.47	0.014 *
12 h	6 [6–6]	5.80 ± 0.41	4 [4–5]	4.48 ± 0.77	1 (1 to 2)	55.0	5.33	<0.001 *
18 h	6 [6–7]	6.20 ± 0.65	5 [5–6]	5.28 ± 0.98	1 (0 to 1)	145.5	3.45	0.001 *
24 h	7 [7–7]	6.92 ± 0.49	6 [5–6]	5.64 ± 0.70	1 (1 to 2)	53.0	5.36	<0.001 *

n = 25 per group, with complete data at every time point; FLACC was assessed at 1, 2, 6, 12, 18, and 24 h postoperatively by personnel blinded to group allocation. Medians with interquartile ranges (25th–75th percentiles), Hodges–Lehmann estimates, z statistics, and p-values are identical to those in Table 3 of the main text. [a] Because FLACC is an ordinal scale, means ± SD are provided for descriptive purposes only; they correspond to the values plotted in Figure 3 of the main text. [b] Hodges–Lehmann estimate of the median difference (dexamethasone minus dexmedetomidine) with a distribution-free 95% CI; because FLACC scores take integer values with many ties, the confidence limits may coincide with the point estimate or with zero. [c] Mann–Whitney U test; z is the absolute value of the standardized test statistic, and p-values are asymptotic and two-sided. * Nominal $p < 0.05$; these exploratory time-point comparisons were not adjusted for multiple comparisons. A longitudinal cumulative-logit ordinal model of FLACC scores could not be validly estimated because of quasi-complete separation of scores between groups (Section 2.7 of the main text).

CI, confidence interval; CPB, cardiopulmonary bypass; FLACC, Face, Legs, Activity, Cry, Consolability; IQR, interquartile range; SD, standard deviation.
